# Depressive Disorder promotes Hepatocellular Carcinoma metastasis via upregulation of ABCG2 gene expression and maintenance of self-renewal

**DOI:** 10.7150/jca.45712

**Published:** 2020-07-09

**Authors:** Hao Hu, Shao-Ju Luo, Zhi-rui Cao, Yingzi Wu, Zhuomao Mo, Yongdan Wang, Ling Yu, Yan Chen, Liang Xu, Shi-Jun Zhang

**Affiliations:** 1 The First Affiliated Hospital, Sun Yat-Sen University, Guangzhou 510080, Guangdong, P. R. China.; 2 Department of Oncology, First Affiliated Hospital of Guangzhou University of Chinese Medicine, Guangzhou (510407), China.; 3 The Sixth Affiliated Hospital, Sun Yat-Sen University, Guangzhou 510080, Guangdong, P. R. China.; 4 Department of Chinese Medicine, the Third Affiliated Hospital, Sun Yat-Sen University, Guangzhou 510630, Guangdong, P. R. China.

**Keywords:** Depressive disorder, Hepatocellular carcinoma, Metastasis, CSCs, ABCG2

## Abstract

Depressive disorder (DD) is the leading cause of disability worldwide and is the most prevalent mood disorder. Accumulative evidence from epidemiological studies has shown that DD is a risk factor for cancer. However, the role and molecular mechanism of DD in hepatocellular carcinoma (HCC) are still unknown. In this study, 30 mice were randomly divided into two groups: the HCC group and the HCC-DD group. The DD mouse model of HCC was established by induction with reserpine every other day and with monthly doses of diethylnitrosamine (DEN). All of the molecular studies were based on primary cell culture, and the effects of DD on HCC cell proliferation and migration and cancer stem cell (CSC) self-renewal were determined by colony formation, wound healing, and sphere culture assays. We found that the CSC markers ABCG2 and CD133 were upregulated in HCC-DD primary cells compared with HCC primary cells. Moreover, HCC-DD primary cells were more aggressive in terms of metastasis and self-renewal than HCC primary cells. Further study revealed that DD promoted tumor growth and metastasis by activating the AKT signaling pathway followed by an increased ABCG2 expression. Taken together, our novel findings indicate that DD promotes proliferation, self-renewal, and metastasis by upregulating ABCG2 in the AKT pathway.

## Introduction

Studies suggest that one out of five people will suffer from a mood disorder during their lifetime 1. Major depressive disorder (MDD) is the leading cause of disability worldwide and is the most prevalent mood disorder 2. Clinical studies report a high prevalence of MDD comorbidity with inflammatory diseases, including cardiovascular diseases, diabetes, metabolic disorders, asthma, and rheumatoid arthritis 3. Depressive disorder(DD), also known as clinical depression, major depression, unipolar depression, or unipolar disorder, it is a mental disorder characterized by an all-encompassing low mood accompanied by experiencing a loss of energy and interest, feelings of guilt, difficulty in concentrating, loss of appetite, and thoughts of death or suicide^4^, ^5^. Depressive disorder is the leading cause of the diminution of health not only in the general population, but also in cancer patients 6. Mental health problems have been shown to have a larger impact on health utility values than physical health problems. To better understand DD, an animal model of the progressive development of depression is needed. In a previous study, repeated administration of reserpine was performed to establish a mouse model of DD 7. Reserpine is an indole alkaloid that has recently been applied as an antihypertensive drug. Reserpine inhibits the uptake of catecholamines by acting as an irreversible inhibitor of the vesicular amine pump and ultimately results in the depletion of catecholamine stores 8. Apart from studying the antidepressive effects of certain novel agents, this model is beneficial for studying the progressive development of depression.

Hepatocellular carcinoma (HCC) is one of the most common primary malignancies and the third most common cause of cancer mortality worldwide 9, 10. The major risk factors for HCC are chronic infection with the hepatitis B or C virus, both of which increase the risk of liver cancer by approximately 20-fold 11, 12. With improvements in epidemiological research, there has been a growing understanding of risk factors that induce hepatocarcinogenesis, but the disease prognosis remains poor. Numerous experimental models have been developed to define the pathogenesis of HCC and have contributed to the current knowledge of HCC. Overall, there are three model types currently used for the study of HCC in mice and rats: chemically induced models, xenograft models, and genetically modified models. The HCC models that are currently used to generally combine two or more model types as liver injuries synergize 13. Two types of carcinogenic compounds, namely, genotoxic and promoting compounds have been used to generate HCC models 14. Chemically induced HCC mouse models mimic the injury-fibrosis-malignancy cycle via the administration of a genotoxic compound alone and, if necessary, a promoting agent 15.

Accumulative evidence has shown that cancer involves dynamic changes in the genome 16. Cancer metastasis is the leading cause of death among patients with any type of malignant tumor 17, and this complex process remains the least understood aspect of cancer biology 18. It is well known that cancer stem cells (CSCs) have the properties of self-renewal, differentiation, and resistance to chemotherapy or radiotherapy 19, 20. Although CSCs represent a small proportion of the tumor cell population, they are key players in tumor initiation, recurrence, and metastasis 21.

DD is an important factor in HCC. Therefore, we aimed to explore the underlying mechanism of DD in HCC progression. In the present study, we hypothesized that DD might function as a promoter of HCC progression. Additionally, we provide evidence that DD is associated with HCC proliferation, self-renewal, and metastasis. We observed AKT pathway activation and upregulation of ABCG2 expression in the HCC-DD group. For the first time, our data demonstrate that DD is a tumor-promoting factor that affects metastasis and self-renewal by upregulating ABCG2 expression.

## Materials and Methods

### Experimental Animals and Chemicals

In total, 30 specific pathogen-free (SPF) C57BL/6 mice (male; weight, 23±2 g) were purchased from the Animal Experimental Center of Guangdong Province. Diethylnitrosamine (N-nitrosodiethylamine, DEN, CAS: 55-18-5) was purchased from Sigma, and reserpine injection was purchased from Aladdin (R101672). All of our studies were approved by the research ethics committee at Sun Yat-Sen University.

### Study Design

All of the C57BL/6 mice were randomly divided into the HCC group and HCC-DD group. In both groups, DEN injections were performed every 30 days to induce HCC development, which took 10 months. The model of HCC mice with depressive disorder (HCC-DD) was established by reserpine administration at 0.1 mg/kg every two days. The body weight and food intake of each mouse were recorded daily. We determined the DD score based on the DD Scale.

### Histochemical Stain

The tissues were embedded in paraffin, and paraffin-embedded tissue samples were cut into 4-5 µm sections. The sections were stained with hematoxylin-eosin (H&E) according to the instructions of the manufacturer. The sections with H&E staining were observed under a light microscope (Olympus Optical, Japan).

### Specimens and i*n vitro* Culture

The tumor tissues were divided from the hepatic tissues in a sterile environment. Fresh tumor tissues were digested with type II collagen (Yeasen, 40508ES60) for 2 h at 37 °C. Cells were desegregated with a 40-μm cell strainer and washed with DMEM. Finally, cells were seeded into culture medium with 20% FBS, 0.1% EGF, 0.1% FGF, 0.1% HGF, 50 units/ml penicillin G and 50 μg/ml streptomycin.

### Cell Viability Assays

Cellular growth curves were plotted by using the cellular viability values assessed by the MTS method. Cells were seeded into 96-well plates at a density of 1 × 10^3^ cells/well in 200 μl of culture medium. At various time points after seeding, the cells in each well were stained with 20 µl of MTS (Promega, G3580) for 3 h at 37 °C, and the OD490 was determined with a microplate reader.

### Cell Colony Formation Assays

For the colony formation assays, 500 cells/2 ml were seeded into a 6-well plate (Corning). The culture medium was subsequently changed every 2 days. After 10 days, the cells were washed with phosphate-buffered saline, fixed with methanol for 15 min at room temperature, and stained with 1% crystal violet for 20 min. The colonies were counted.

### Sphere-Formation Assay

Cells were cultured in DMEM/F12 medium containing 20 ng/ml bFGF, 20 ng/ml EGF and B27 supplement (Invitrogen) on 6-well low-attachment plates (Corning, Acton, MA, USA) at a density of 10,000 cells/well. Under these conditions, the cells grew in suspension as spherical clusters, and the conditioned medium was changed every 3-4 days. After incubation at 37 °C for 7-10 days, pictures were taken under a microscope, and the number of spheres was counted in all wells.

### Real-Time Quantitative PCR Analysis

The expression level of the gene was determined by RT-PCR. Total mRNA of cells was isolated using TRIzol reagent (Invitrogen, CA, USA). The samples were subjected to reverse transcription using a cDNA Synthesis Kit (Thermo, K1622). The internal control in our study to measure the gene expression level was GAPDH. The relative expression levels of the target genes were estimated by two power values of ΔCt (the Ct of GAPDH minus the Ct of the target gene), and the experiments were repeated three times. The sequences of the primer sets are shown in Table 1.

### Western Blot Analysis

Cells were lysed in RIPA buffer containing a protease (TargetMol, CC0001) and phosphatase (TargetMol, CC0004). The primary antibodies against vimentin (#5741), N-cadherin (#13116), fibronectin/FN1 (#26836), E-cadherin (#14472), DSP (#5885), GAPDH (#5174), PTEN (#9188), AKT (#4691), and p-AKT (#4060) were obtained from Cell Signaling Technology. The secondary antibodies were HRP-conjugated goat anti-rabbit or anti-mouse antibodies (1:10,000, Proteintech).

### Wound-Healing Assays

Cells were digested and seeded in a 6-well plate. A scratch wound assay was performed by generating a wound in the center of each well in a 6-well plate with a sterile 200 µl pipette tip. The unattached cells were removed by washing with PBS, and serum-free medium or medium with 3% FBS was added. Subsequently, cells were observed with an inverted microscope at 0, 20 or 40 h.

### Statistical Analysis

All data in this study were evaluated with SPSS 21.0 software (SPSS Inc., Chicago, USA) and GraphPad Prism (GraphPad software). All data are shown as the mean ± standard deviation. The results of real-time quantitative PCR were evaluated using Student's t-test. *p*<0.05 indicated a statistically significant difference.

## Results

### Establishment of the HCC-DD model with DEN and reserpine

**Figure 1:**
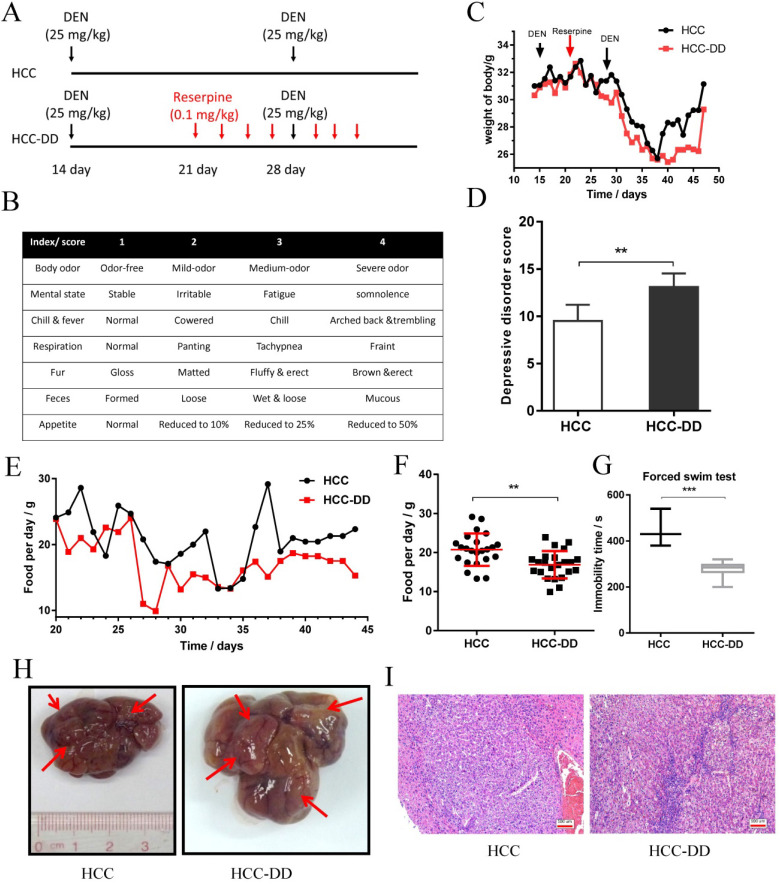
** Establishment of HCC-DD model with DEN and reserpine.** (**A**) Mice received 25 mg/kg DEN on the 14th and 30th days and were then induced by reserpine every two days starting on day 21. (**B**) The table was used to identify depressive disorder by determining scores. (**C**) The growth curve of the mice. (**D**) Depressive disorder score of the HCC and HCC-DD groups; (**E, F**) Food intake curve for every single day (**G**) The time of immobility in water during the forced swim test. (**H**) Liver tumor image of mouse model. (**I**) HE staining of the liver tissue. **p < 0.01, ***p < 0.005.

First, mice received a single injection of 25 mg/kg DEN. Afterward, mice were treated with reserpine (0.1 mg/kg) every two days starting on the 21st day. Then, all the mice were intraperitoneally injected with 25 mg/kg diluted DEN on the 30th day (Figure 1A). The DD rating scale is an important method for assessing behavioral depression, and it was used to identify DD by evaluating scores for factors including body odor, mental state, chill & fever, respiration, fur, feces, and appetite (Figure 1B). The DD assessment showed that the HCC-DD group had higher scores than the HCC group (p<0.01) (Figure 1D). Compared with those in the HCC group, the mice in the reserpine-treated HCC-DD group did not significantly differ in terms of body weight during the first 38 days but had a reduction in body weight at day 38 (Figure 1C).

The food intake curve showed the food preference difference between the HCC-DD and HCC groups (Figure 1E). The diagram showed that the HCC-DD group consumed less food per day than the HCC group (Figure 1F). The forced swim test revealed that the HCC-DD group had a lower immobility time (Figure 1G). These results indicated that DD was successfully established. The liver tissues in all groups were analyzed at the 40th week. Photographs of the liver showed that both groups had tumor formation (Figure 1H). Hematoxylin-eosin (HE) staining of the liver tissues from the HCC group and HCC-DD group showed that the carcinoma cells were of all sizes, with some multinucleated giant tumor cells, and funicular slices were distributed and accumulated irregularly without normal hepatocyte construction (Figure 1I). To some extent, the results indicated that the degree of malignancy of the tumor tissue was higher in the HCC-DD group than in the HCC group.

### Depressive disorder promotes cell growth colony formation *in vitro*

**Figure 2:**
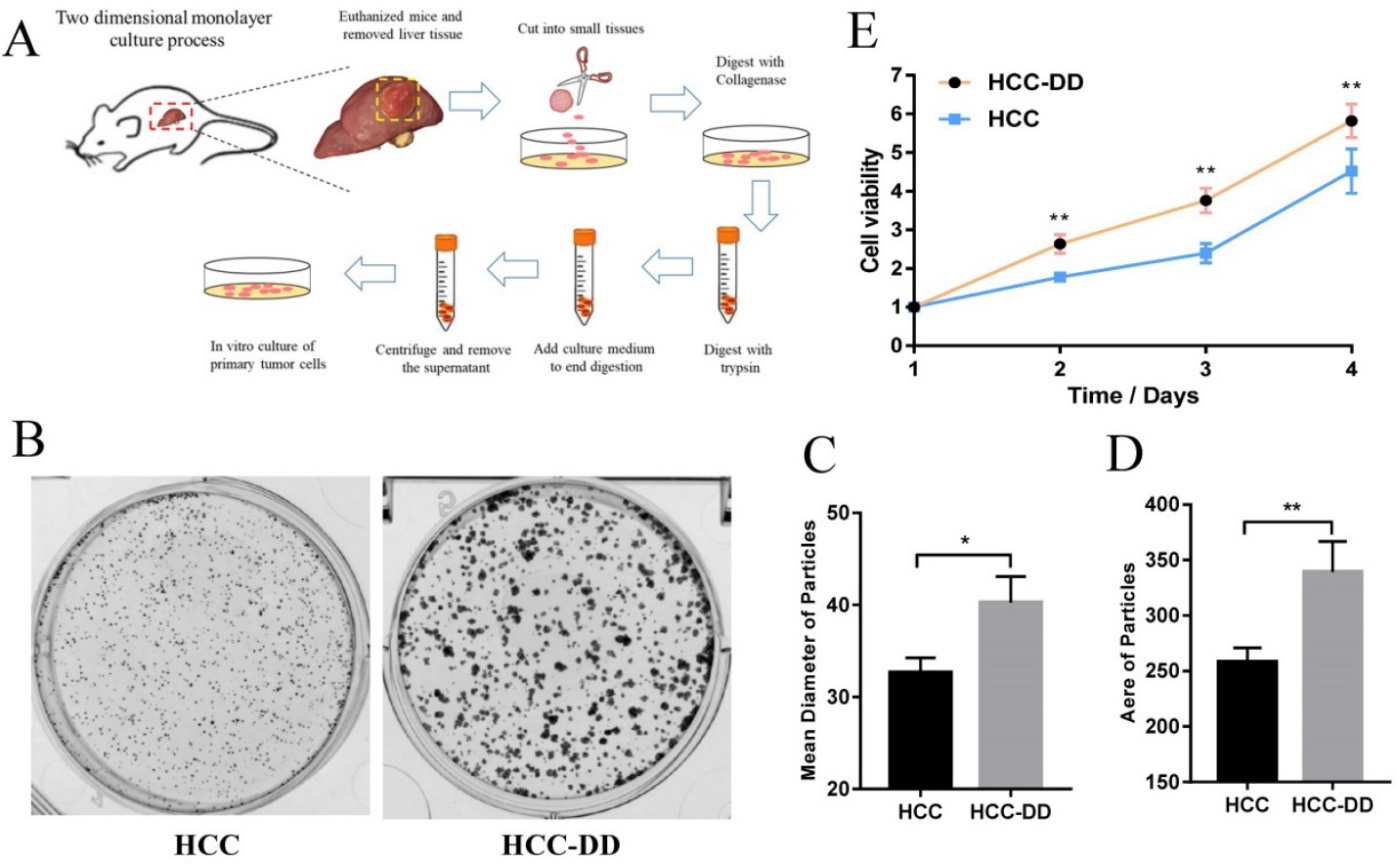
** Depressive disorder promotes cell growth colony formation *in vitro*.** (**A**) Flow chart of primary culture in a mouse model. (**B, C, D**) Representative micrographs of colony formation. (**E**) The growth curve of the primary cell line. The data represent the mean ± S.D., n=3; *p < 0.05, **p < 0.01.

Primary culture is the most useful in vitro system and represents an important tool for cancer research. We carried out primary culture of HCC tumor tissues from the mouse model, cultured them as a monolayer of tumor cells, and then performed functional assays (Figure 2A). To determine more effects of DD in HCC, we performed colony formation and proliferation assays. We observed that HCC-DD cells had a better cell colony formation ability than HCC cells (Figure 2B). There were also differences in the mean diameter and the area of the particles between the two cell lines (Figure 2C-D). The cell growth curve showed that DD increased cell proliferation (Figure 2E).

### Depressive disorder upregulates ABCG2 and promotes self-renewal in an HCC mouse model

**Figure 3:**
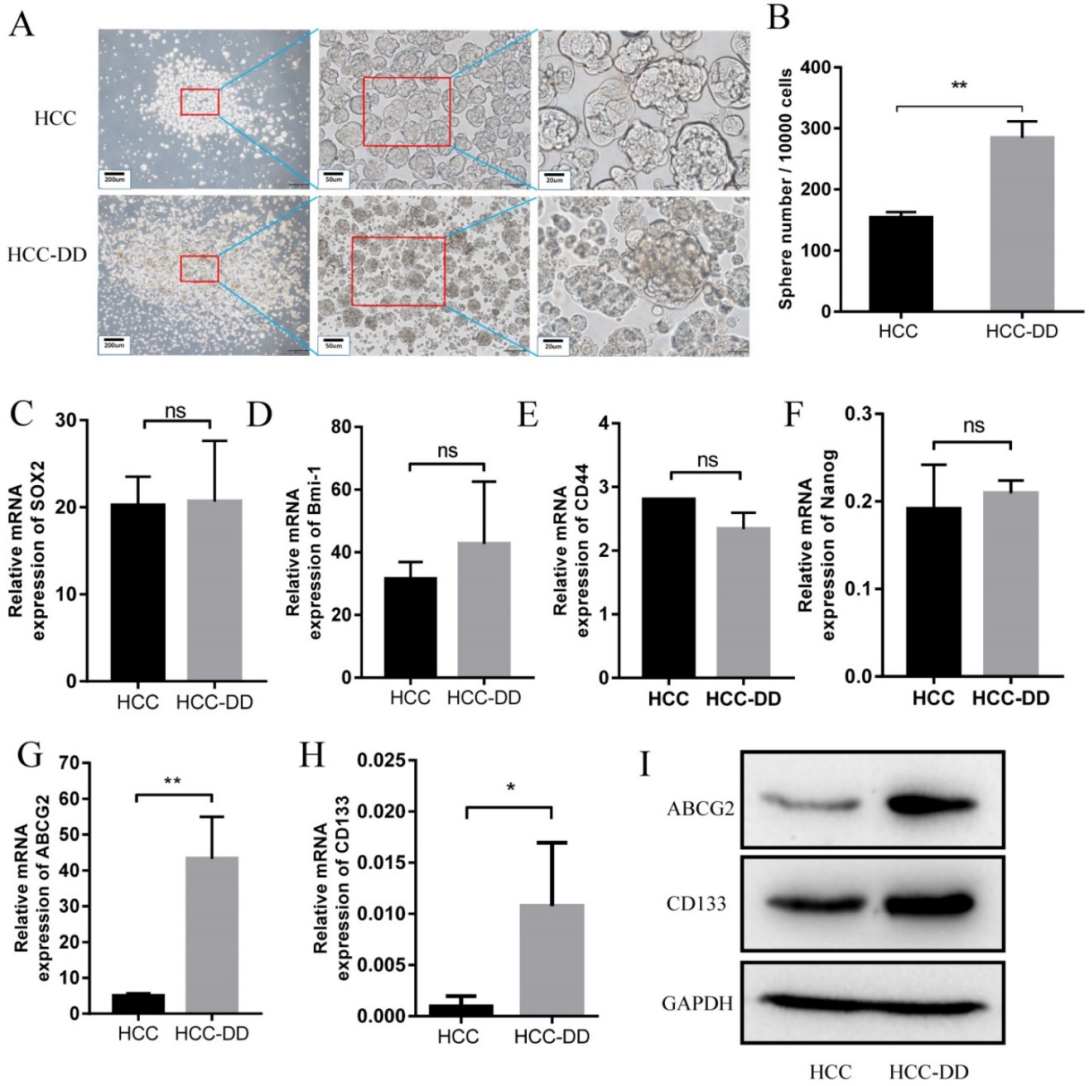
** Depressive disorder upregulates ABCG2 and promotes self-renewal in the HCC mouse model.** Representative micrographs of sphere culture (**A**) and the quantitative figure of sphere number (**B**). (**C-G**) The mRNA expression of related stem cell markers in HCC and HCC-DD primary culture cells. (**H**) Western blotting of ABCG2 and CD133 in HCC and HCC-DD primary culture cells.

HE staining showed that the HCCs in the HCC-DD group were more malignant than those in the HCC group. To determine the underlying mechanisms of DD in the HCC mouse model, we established primary cell lines of liver tumors from HCC and HCC-DD mice. To examine the effect of DD on maintaining CSC characteristics, we performed a sphere culture assay, and the results showed that the HCC-DD cell line had a larger tumorsphere size and a higher tumorsphere number than the HCC cell line (Figure 3A and 3B). These data revealed that DD could promote self-renewal in some way. To determine the detailed mechanisms, we examined stem cell markers, and there was no significant difference in the expression of SOX2, Bmi-1, CD44, or Nanog between the two cell lines (Figure 3C-F). RT-qPCR showed that ABCG2 and CD133 were highly expressed in the HCC-DD group (Figure 3G-H), and Western blotting showed similar results (Figure 3I). The in vitro assays revealed that DD could specifically upregulate the stem cell markers ABCG2 and CD133 and then promote self-renewal.

### Depressive disorder promotes metastasis in an AKT-dependent manner

**Figure 4:**
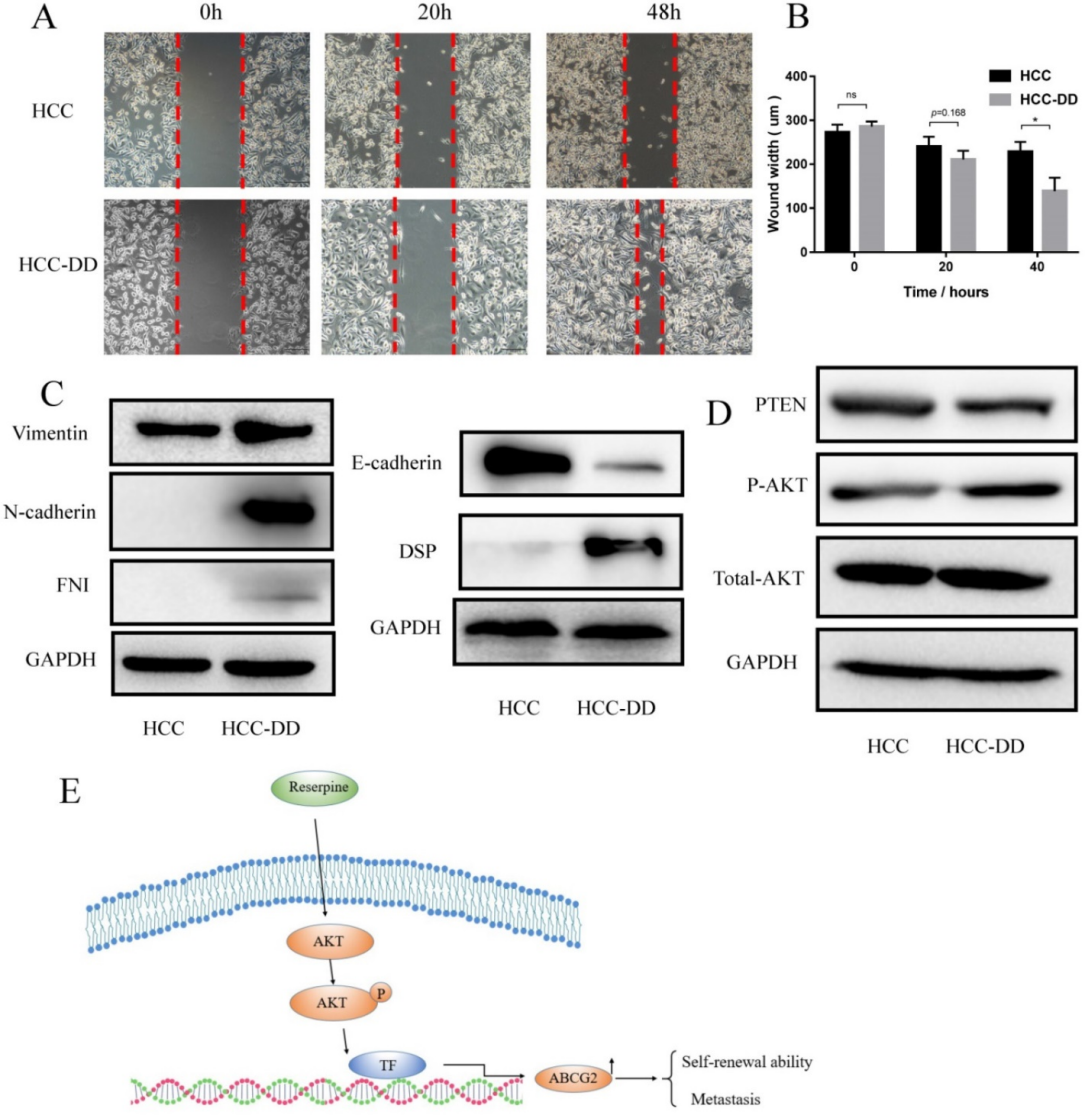
** Depressive disorder promotes metastasis in an AKT-dependent manner.** (**A**) The migration ability of cells in the HCC-DD group was dramatically increased, as determined by a wound-healing assay. (**B**) Quantification of the wound-healing assay. (**C**) Western blotting of EMT markers in HCC and HCC-DD primary cell cultures. (**D**) PTEN, AKT, and p-AKT levels in HCC and HCC-DD primary culture cells. Schematic illustration of how reserpine affects the AKT pathway, regulates ABCG2, and then promotes metastasis and CSC function (**E**).

Multiple studies have suggested that CSCs serve as the basis of metastasis. Since HCC-DD cells have high self-renewal ability, we hypothesized that DD might also affect metastasis. To verify this hypothesis, we performed a wound-healing assay. The results showed that HCC-DD cells had a higher wound-healing rate than HCC cells (Figure 4A). A difference in the wound width became evident at 40 h (Figure 4B). To determine whether DD affects the epithelial-mesenchymal transition (EMT) process, we examined EMT markers by Western blotting. HCC-DD cells exhibited increased levels of mesenchymal markers, such as vimentin, N-cadherin, and FNI, and decreased levels of the epithelial proteins E-cadherin and desmoplakin (Figure 4C). These results proved that DD could promote EMT in vitro. To determine which pathway might be involved, we examined the literature and found that HCC metastasis is mostly associated with activation of the AKT pathway, so we evaluated AKT, p-AKT and PTEN expression (Figure 4D). Interestingly, we found that PTEN expression was decreased in HCC-DD cells compared with HCC cells. The results showed that phosphorylation of AKT was increased, indicating that the AKT pathway was activated after DD occurred in the HCC mouse model.

## Discussion

Mood disorders are associated with persistently high morbidity and mortality rates, despite the widespread availability of antidepressant treatments. They are ranked by the World Health Organization (WHO) as the main global cause of “years of life lived with disability” for all age groups 22. Clinical features include depressed mood, loss of energy and interest, feelings of guilt, difficulty concentrating, loss of appetite, and thoughts of death or suicide. Some studies have found an etiological association between DD and the risk of liver disease 23 and HCC 24. However, the underlying mechanism of DDs following HCC remains unknown.

Reserpine is used to establish mouse depression models 25. Reserpine is not lethal, and a chronic rather than an acute dose of reserpine was used in this study to induce DD in mice. In our study, we based our model on the HCC mouse model and then treated the mice with reserpine every two days. Compared with the HCC group, the HCC-DD group did not significantly differ in terms of body weight over the first 38 days, but there was a reduction in body weight on the 38th day, which showed that as time passed, reserpine affected the body weights of HCC mice. To some extent, DD can increase the degree of malignancy. In our analysis, we determined the scores for each factor in the DD rating scale and found that the DD scores were different between the two groups. Food intake and immobility time were considerably different between the HCC-DD and HCC groups. HE staining showed that the degree of malignancy of the tumor tissue was higher in the HCC-DD group than in the HCC group. These results suggested that HCC-DD mouse models were successfully established after reserpine administration.

Primary culture is a crucial method in the study of HCC in vitro. One important advantage is that the heterogeneity of the cell populations composing a primary culture partially reproduces the tumor microenvironment and crosstalk between malignant and healthy cells, neither of which is possible with cell lines 26. For this reason, primary culture has become the most useful in vitro system and represents an important tool for cancer research. To determine the underlying mechanism of DD in the HCC mouse model, we performed primary culture of mouse liver tumor tissues. The proliferation assay showed that cell proliferation was significantly increased in the HCC-DD group (Figure 2C) compared with the HCC group, indicating that DD promoted tumor growth in the HCC-DD model.

It is well known that CSCs have the properties of self-renewal and differentiation. Specific markers are present in stem cells. For example, there are several biomarkers for CSCs, including SOX2, Bmi1, CD44, Nanog, and ABCG2 27, 28. CD133 is a specific CSC marker in HCC. Sphere culture assays showed that the HCC-DD cell line had a larger tumorsphere size and tumorsphere number than the HCC cell line. This result indicated that DD might be associated with the self-renewal of CSCs, and further experiments showed that DD can upregulate ABCG2 and CD133 expression in vitro. ABCG2 and CD133 are two typical CSC markers for HCC, and ABCG2 is a marker of the CSC side population (SP) 29. The SP phenotype is also a widely used marker for CSCs, and it is associated with drug resistance. These findings revealed that DD can promote the CSC function by upregulating ABCG2 in the HCC mouse model.

Multiple studies have suggested that CSCs serve as the basis of metastatic dissemination, and CSCs may directly or indirectly contribute to the generation of metastases 30. To verify the correlation between DD and CSCs or metastasis, we carried out the wound-healing assays and EMT marker tests. The results showed that DD promoted wound healing, increased the levels of mesenchymal markers and decreased the levels of epithelial markers. These results indicated that DD promotes the EMT process in HCC. The AKT pathway plays a pivotal role in fundamental cellular functions such as cell proliferation and survival by phosphorylating a variety of substrates. The results showed that phosphorylation of AKT was increased, which means that the AKT pathway was activated in HCC-DD cells; this suggests that DD can promote AKT activation.

In conclusion, we found that DD plays a strong role in regulating HCC growth, self-renewal, and metastasis in an HCC mouse model. In addition, at the molecular level, the CSC markers ABCG2 and CD133 were upregulated, and the AKT pathway was activated in the HCC-DD group. DD also promotes the EMT process. Our novel findings are summarized in Figure 4E. These significant findings revealed that DD promotes HCC metastasis by upregulating ABCG2 gene expression and maintaining self-renewal in an AKT-dependent manner. However, further studies are needed to explore the relationship between ABCG2 and the AKT pathway in the DD process in HCC.

## Figures and Tables

**Table 1:** The sequences of the primer

Genes	Primer	Sequences
SOX2	Forward	AACGGCAGCTACAGCATGATGC
SOX2	Reverse	CGAGCTGGTCATGGAGTTGTAC
Bmi-1	Forward	ACTACACGCTAATGGACATTGCC
Bmi-1	Reverse	CTCTCCAGCATTCGTCAGTCCA
CD44	Forward	CGGAACCACAGCCTCCTTTCAA
CD44	Reverse	TGCCATCCGTTCTGAAACCACG
Nanog	Forward	GAACGCCTCATCAATGCCTGCA
Nanog	Reverse	GAATCAGGGCTGCCTTGAAGAG
ABCG2	Forward	CAGTTCTCAGCAGCTCTTCGAC
ABCG2	Reverse	TCCTCCAGAGATGCCACGGATA
CD133	Forward	CTGCGATAGCATCAGACCAAGC
CD133	Reverse	CTTTTGACGAGGCTCTCCAGATC
GAPDH	Forward	CATCACTGCCACCCAGAAGACTG
GAPDH	Reverse	ATGCCAGTGAGCTTCCCGTTCAG

## Abbreviations

HCC: Hepatocellular carcinoma
DD: Depressive disorder
MDD: Major depressive disorder
DEN: Diethylnitrosamine
CSC: Cancer stem cell
SPF: Specific pathogen-free
EMT: Epithelial-mesenchymal transition
